# ﻿Three new species of the leafhopper genus *Arboridia* Zachvatkin (Hemiptera, Cicadellidae, Typhlocybinae), with a key and checklist to known species of China

**DOI:** 10.3897/zookeys.1196.118829

**Published:** 2024-03-28

**Authors:** Chang Han, Bin Yan, Xiaofei Yu, Maofa Yang, Michael D. Webb

**Affiliations:** 1 Institute of Entomology, Guizhou Provincial Key Laboratory for Agricultural Pest Management of the Mountainous Region, Guizhou University, Guiyang 550025, China; 2 College of Tobacco Science, Guizhou University, Guiyang 550025, China; 3 Department of Entomology, The Natural History Museum, Cromwell Road, London, SW7 5BD, UK

**Keywords:** *Arboridia*, Hemiptera, identification key, new species, taxonomy, Typhlocybinae

## Abstract

Three new species of the leafhopper genus *Arboridia*Zachvatkin 1946, Arboridia (Arboridia) furcata Han, **sp. nov.**, Arboridia (Arboridia) rubrovittata Han, **sp. nov.**, and Arboridia (Arboridia) robustipenis Han, **sp. nov.**, are described and illustrated from fruit trees in Southwest China. A key and checklist to known species from China are provided.

## ﻿Introduction

The leafhopper genus *Arboridia* Zachvatkin, 1946 belongs to the tribe Erythroneurini of the subfamily Typhlocybinae (Hemiptera: Auchenorrhyncha: Cicadellidae) and includes two subgenera, *Arboridia* Zachvatkin, 1946 and *Arborifera* Sohi & Sandhu, 1971. Species feed on a variety of plants including fruit trees, hawthorn, maple, honeysuckle, dogwood and several other plants (Song and Li 2013). So far, 84 species have been described in this large genus, distributed throughout the Palaearctic and Oriental regions, including 25 valid species from China (Song et al. 2016; Cao et al. 2019).

In this study, three new species are described from Guizhou, China. Photographs of the whole body, illustrations of male genitalia, and biological information such as host plants and distributional records are provided. In addition, an updated key to the *Arboridia* species from China is provided.

## ﻿Materials and methods

Specimens used in this study were collected from grape, kiwi and walnut trees in Guizhou, China using a sweep net. A Nikon SMZ 1270 microscope was used to dissect the specimens and an Olympus CX41 microscope for observing and drawing the male genitalia. A KEYENCE VHX-6000 digital microscope was used to take pictures of the male habitus. Morphological terminology used in this work follows Dietrich (2005) and Dworakowska (1993). All specimens examined in this study are deposited in the Institute of Entomology, Guizhou University, China (GUGC).

## ﻿Taxonomy

### ﻿ Arboridia

Taxon classificationAnimaliaHemipteraCicadellidae

Zachvatkin

10B21056-0E40-5299-9C9C-DF22F2FF38DB

Arboridia Zachvatkin, 1946: 153. Type species. Typhlocybaparvula Boheman, 1845.
Khoduma Dworakowska, 1972: 403. Synonymised by Dworakowska and Viraktamath (1975: 529). Type species. Khodumajacobii Dworakowska, 1972.

#### Diagnosis.

Head slightly narrower than pronotum, crown weakly produced with fore margin rounded. Head and thorax yellow; vertex usually with pair of dark subapical spots; pronotum usually with irregular brown symmetric markings; scutellum with brown basal triangles. Forewing either without marking, with oblique vittae or with dark spots. Ventral abdominal apodemes small and extended to or beyond posterior margin of 3^rd^ sternite. Male pygofer with widespread microtrichia and several small rigid setae on inner surface of hind margin; dorsal appendage present, free from pygofer side; ventral appendage absent; phragma lobe with spine-like setae present on each side of aedeagus, attached to dorsal apodeme of aedeagus by ligaments (Fig. 51). Subgenital plate upturned apically with lateral margin basally expanded triangular shaped with 2–4 lateral macrosetae in an oblique row slightly basad of midlength; lateral margin with short spine-like setae. Style apex usually with 3 points, sometimes 2^nd^ point absent. Aedeagus with shaft laterally compressed, usually with processes, gonopore apical on ventral surface; dorsal apodeme and preatrium present or absent. Connective U- or V- shaped with median anterior lobe absent.

#### Distribution.

Palaearctic and Oriental regions.

##### ﻿Checklist of Chinese species of *Arboridia*

1. *Arboridiaagrillacea* (Anufriev, 1969b: 182–183, fig. 13: 1–6, *Erythroneura*); Anufriev, 1978a: 87, transferred to *Arboridia*; Song & Li, 2013: 243–244, figs J, j, jj, 63–69; *Arboridiakoreana* Oh & Jung, 2015: 447–448, figs 1, 3, 5, 7, 9–15, synonym. Distribution: Gansu, Guangxi, Guizhou, Henan, Shaanxi, Sichuan.

2. *Arboridiaanteoculara* Song & Li, 2013: 230–233, figs A, a, 1–7. Distribution: Guizhou.

3. *Arboridiaapicalis* (Nawa, 1913a: 480–486, Pl. 24, *Zygina*); Cockerell, 1920a: 247, *Erythroneura*; *Erythroneurasandagouensis* Vilbaste, 1968a: 108, synonym; Anufriev, 1969b: 185–186, fig. 15: 8–13; Dworakowska, 1970g: 607–608, fig. 18, transferred to *Arboridia*. Distribution: Anhui, Guizhou, Hebei, Henan, Hubei, Jiangsu, Liaoning, Shannxi, Shandong, Taiwan, Zhejiang.

4. *Arboridiabaiyunensis* Song & Li, 2013: 233–234, figs B, b, 8–14. Distribution: Henan.

5. Arboridia (Arborifera) changlingensis Jiang, Luo & Song, 2021: 354–355, figs 5–8, 27–34. Distribution: Guizhou.

6. *Arboridiacincta* Song & Li, 2015: 585–587, figs A–C, 1–7. Distribution: Henan.

7. *Arboridiacuihuashana* Song & Li, 2013: 237–238, figs E, e, 29–35. Distribution: Shaanxi.

8. *Arboridiaechinata* Song & Li, 2013: 239–240, figs G, g, gg, 42–48. Distribution: Guizhou.

9. *Arboridiafurcata* Han, sp. nov. Distribution: Guizhou.

10. *Arboridiahuajiangensis* Jiang, Luo & Song, 2021: 351–353, figs 1–4, 9–26. Distribution: Guizhou.

11. *Arboridiajinghongensis* Pu, Wang & Song, 2023: 296–297, figs 1a–f, 2a–h. Distribution: Yunnan.

12. *Arboridiakakogawana* (Matsumura, 1932: 113, *Zygina*); Ishihara, 1953b: 33, *Erythroneura*; Dworakowska, 1970g: 610, figs 25–29, transferred to *Arboridia*. Distribution: Beijing, Guizhou, Shandong, Xinjiang.

13. *Arboridialunula* Song & Li, 2013: 234–236, figs D, d, 22–28. Distribution: Guizhou.

14. *Arboridialuojiashangensis* Zhang, Jiang & Song, 2022: 6–8, figs 21–32. Distribution: Guizhou.

15. *Arboridiamaculifrons* (Vilbaste, 1968a: 107, *Erythroneura*); Dworakowska, 1970g: 611, figs 19–22, transferred to *Arboridia*. Distribution: Guizhou, Hebei.

16. *Arboridiaochracea* Song & Li, 2015: 587–588, figs D–F, 8–15. Distribution: Henan.

17. *Arboridiaparaprocessa* Song & Li, 2013: 239, figs F, f, 36–41. Distribution: Guizhou, Henan.

18. *Arboridiareniformis* Song & Li, 2013: 234, figs C, c, cc, 15–21. Distribution: Yunnan.

19. *Arboridiaremmi* (Vilbaste, 1968a: 103, *Erythroneura*); Anufriev, 1969b: 183–184, figs 15: 1–7; Dworakowska, 1970g: 613, transferred to *Arboridia*. Distribution: Guizhou.

20. *Arboridiarobustipenis* Han, sp. nov. Distribution: Guizhou.

21. *Arboridiarubrovittata* Han, sp. nov. Distribution: Guizhou.

22. *Arboridiasinensis* Guglielmino, Xu, Buckle & Dong, 2012: 878–881, figs 1: A–F, 2: A–B. Distribution: Yunnan.

23. *Arboridiasuputinkaensis* (Vilbaste, 1968a: 109, *Erythroneura*); Dworakowska, 1970g: 613, transferred to *Arboridia*. Distribution: Henan, Zhejiang. https://hoppers.speciesfile.org/otus/43920/overview (Dmitriev et al. 2022)

24. Arboridia (Arborifera) surstyli Cai & Xu, 2006: 75–76, figs 1: 1–10. Distribution: Henan, Zhejiang.

25. *Arboridiasuzukii* (Matsumura, 1916b: 396, *Zygina*); Ishihara, 1953b: 34, *Erythroneura*; *Erythroneuraarboricola* Vilbaste, 1968a: 101, synonym; Dworakowska, 1970g: 613, transferred to *Arboridia*. Distribution: Gansu, Guizhou, shannxi, shanxi, Taiwan. https://hoppers.speciesfile.org/otus/43922/overview.

26. *Arboridiatridentata* Song & Li, 2013: 240–241, figs H, h, 49–55. Distribution: Yunnan.

27. *Arboridiaxiaotungensis* Zhang, Jiang & Song, 2022: 2–5, figs 1–20. Distribution: Guizhou.

28. *Arboridiazhenyuana* Song & Li, 2013: 242–243, figs I, i, 56–62. Distribution: Gansu.

### ﻿Key to species (males) of *Arboridia* species from China

(modified from Jiang et al. 2021)

**Table:** 

1	Preatrium of aedeagus short or absent (*Arborifera*)	**2**
–	Preatrium of aedeagus well developed (Figs 24, 39, 55) (*Arboridia*)	**3**
2	Aedeagal shaft with pair of sharp inverted processes on dorsal margin	***A.surstyli***
–	Aedeagal shaft with one broad triangular process on dorsal margin	***A.changlingensis***
3	Aedeagus without process, shaft with pair of lateral flanges	**4**
–	Aedeagus with processes, shaft without pair of lateral flanges	**6**
4	Aedeagal shaft with lateral flanges serrate	***A.zhenyuana***
–	Aedeagal shaft with lateral flanges not serrate	**5**
5	Aedeagal shaft with lateral flanges narrow, entire	***A.agrillacea***
–	Aedeagal shaft with larger lateral flanges partly wrapped around shaft	***A.jinghongensis***
6	Aedeagus with one process	**7**
–	Aedeagus with more than one process	**9**
7	Aedeagus with process arising from preatrium	***A.apicalis***
–	Aedeagus with process arising from midlength of shaft	**8**
8	Aedeagus with dorsal apodeme extremely expanded in lateral view	***A.sinensis***
–	Aedeagus with dorsal apodeme narrow in lateral view	***A.tridentata***
9	Aedeagus with one or two pairs of processes	**10**
–	Aedeagus with three or more processes	**19**
10	Aedeagal shaft with two pairs of processes, one at apex and one at base	***A.ochracea***
–	Aedeagal shaft with one pair of processes arising from apex or base	**11**
11	Aedeagus with processes arising from base of shaft	**12**
–	Aedeagus with processes arising from apex of shaft	**15**
12	Aedeagus with two pairs of basal processes	***A.anteoculara***
–	Aedeagus with one pair of basal processes	**13**
13	Aedeagus with processes slender and bent basad apically (Figs 17, 24)	***A.furcata* sp. nov.**
–	Aedeagus with processes stout and straight	**14**
14	Aedeagus with dorsal apodeme narrow in lateral view	***A.lunula***
–	Aedeagus with dorsal apodeme extremely expanded in lateral view	***A.maculifrons***
15	Aedeagus with apical processes directed basally	**16**
–	Aedeagus with apical processes directed distally	**18**
16	Apex of aedeagal shaft acuminate in ventral view	***A.cincta***
–	Apex of aedeagal shaft truncate in ventral view	**17**
17	Aedeagus without subapical bifurcation in ventral view	***A.reniformis***
–	Aedeagus with subapical bifurcation in ventral view	***A.xiaotungensis***
18	Aedeagal shaft without spines	***A.cuihuashana***
–	Aedeagal shaft with numerous short spines	***A.echinata***
19	Aedeagal shaft with two or three processes at midlength	**20**
–	Aedeagal shaft with one pair of apical processes	**22**
20	Aedeagal shaft with three processes subbasally, a pair of upper bifurcate processes and a slightly more ventral process (Figs 35, 40)	***A.rubrovittata* sp. nov.**
–	Aedeagal shaft with two processes at midlength	**21**
21	Aedeagal shaft with two processes fused for 2/3 of their length (Figs 50–51, 55–56)	***A.robustipenis* sp. nov.**
–	Aedeagal shaft with two processes one above the other	***A.luojiashangensis***
22	Apical processes of aedeagal shaft directed basally	**23**
–	Apical processes of aedeagal shaft directed distally	**24**
23	Aedeagal shaft with slender apical processes, without ventral protrusion	***A.suputinkaensis***
–	Aedeagal shaft with short apical processes, with small ventral protrusion	***A.huajiangensis***
24	Preatrium of aedeagus longer than shaft	**25**
–	Preatrium of aedeagus shorter than shaft	**26**
25	Aedeagal shaft with numerous short spines	***A.suzukii***
–	Aedeagal shaft without spines	***A.remmi***
26	Aedeagal shaft with distinct extension at midlength	***A.baiyunensis***
–	Aedeagal shaft without extension at midlength	**27**
27	Aedeagus with dorsal apodeme and shaft narrow in lateral view; preatrium with a long ventral process	***A.paraprocessa***
–	Aedeagus with dorsal apodeme and shaft expanded in lateral view; preatrium with a short ventral process	***A.kakogawana***

### ﻿ Arboridia (Arboridia) furcata

Taxon classificationAnimaliaHemipteraCicadellidae

Han sp. nov.

85DDE2C5-143E-5095-A6B6-D8179037741E

https://zoobank.org/92B51DA2-4F3F-40E6-9F01-D8D34BAD4884

Figs 1–4 , 14–20 , 21–28

#### Description.

**Figures 1–13.**
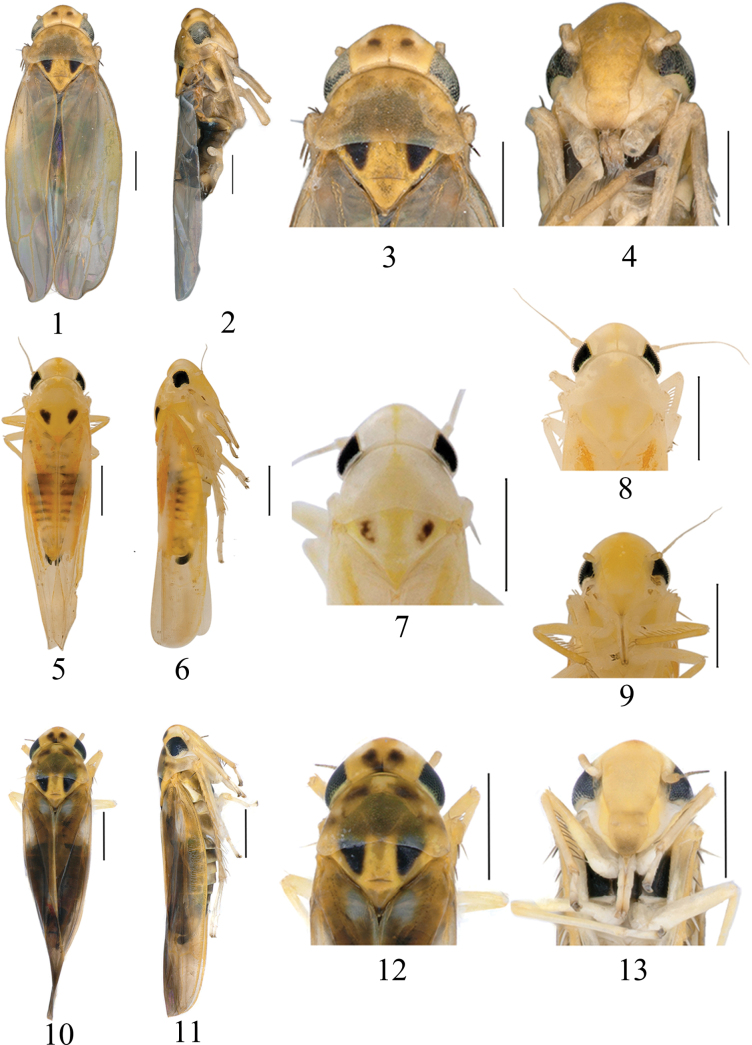
External morphology of *Arboridia* species **1–4***Arboridiafurcata* Han, sp. nov. **1** habitus, dorsal view **2** habitus, lateral view **3** head and thorax, dorsal view **4** face **5–9***Arboridiarubrovittata* Han, sp. nov. **5** habitus, dorsal view **6** habitus, lateral view **7, 8** head and thorax, dorsal view **9** face **10–13***Arboridiarobustipenis* Han, sp. nov. **10** habitus, dorsal view **11** habitus, lateral view **12** head and thorax, dorsal view **13** face. Scale bars: 0.5 mm.

**Figures 14–20.**
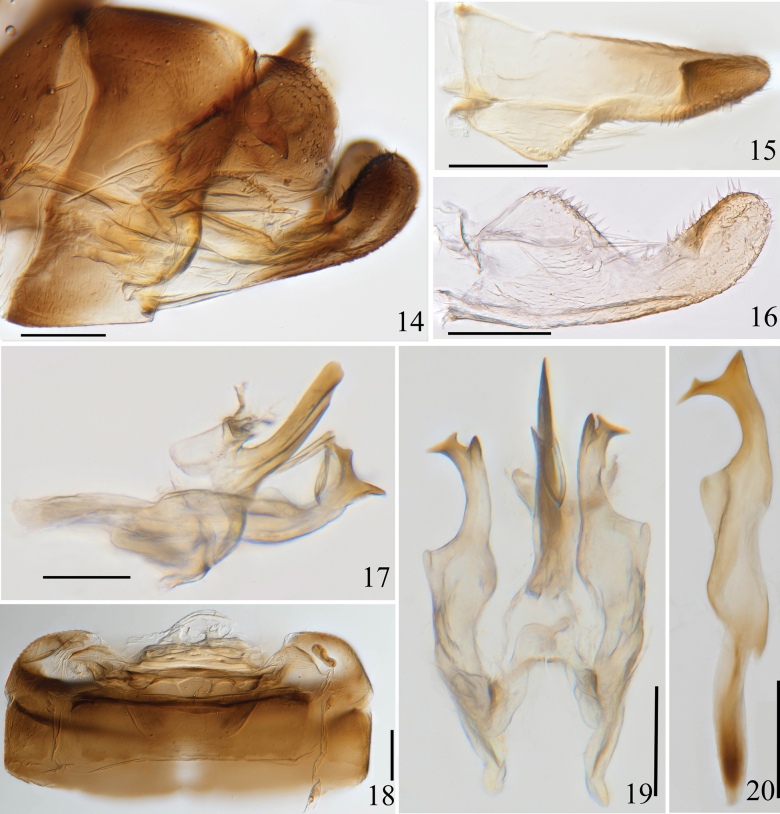
Arboridia (A.) furcata Han, sp. nov. **14** male genitalia, lateral view **15** subgenital plate, dorsal view **16** subgenital plate, ventral view **17** aedeagus, connective and style, lateral view **18** abdominal apodemes **19** aedeagus, connective and style, ventral view **20** style, lateral view. Scale bars: 0.1 mm.

Dorsum yellowish brown; eyes grey with posterior margin beige; vertex with a pair of black spots subapically; coronal suture indistinct distally, pale brown basally (Figs 1–3). Face yellowish brown with median area of frontoclypeus and anteclypeus brighter towards apex; lorum and gena whitish (Fig. 4). Pronotum yellowish brown with brownish spots at anterior margin. Scutellum yellow with lateral triangles dark brown (Fig. 3). Forewing hyaline, veins brown. Abdominal tergites black; sternites milky white; subgenital plate dark apically (Figs 2, 14).

Ventral abdominal apodemes small, extended to 4^th^ sternite (Figs 18, 27).

**Figures 21–28.**
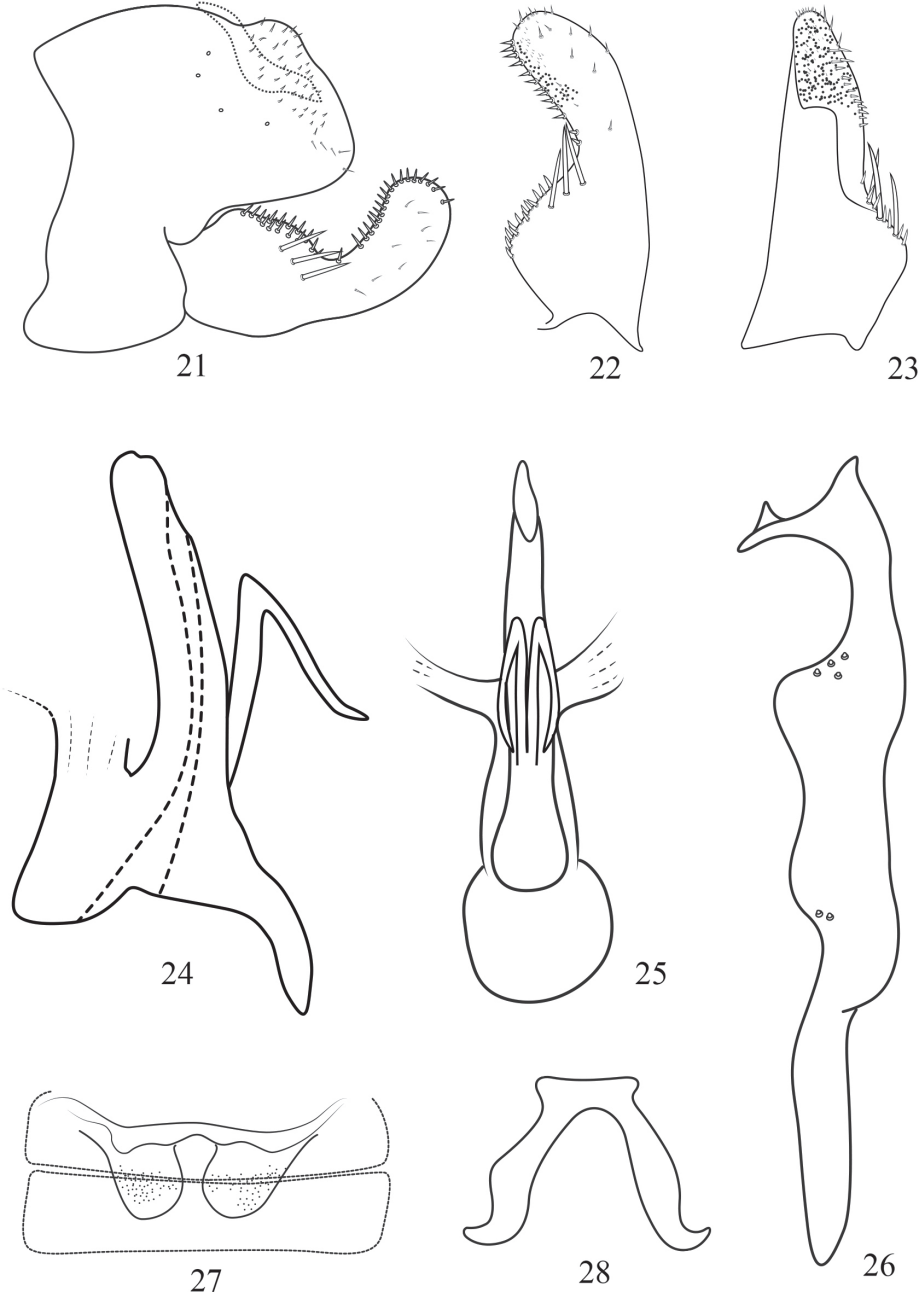
Arboridia (A.) furcata Han, sp. nov. **21** male pygofer, lateral view **22** subgenital plate, ventral view **23** subgenital plate, dorsal view **24** aedeagus, lateral view **25** aedeagus, ventral view **26** style, lateral view **27** abdominal apodemes **28** connective.

***Male genitalia*.** Pygofer dorsal appendage simple, slender and wavy, with the apex obliquely truncate (Figs 14, 21). Subgenital plate with 3 lateral macrosetae in an oblique row slightly basad of midlength laterally (Figs 15, 16, 22, 23). Style long and slender, apex with 3 points; preapical lobe well developed; several small tubercles subapically and at midlength (Figs 20, 26). Aedeagal shaft long and stout, slightly laterally compressed, a pair of long slender basal processes on ventral surface of the shaft, parallel to the shaft in their basal half, then sharply turned in proximal direction in their distal half (Figs 17, 19, 24, 25); dorsal apodeme short and robust, expanded laterally at apex; preatrium short (Figs 17, 24). Connective U-shaped, with lateral arms long and stem broad (Figs 19, 28).

#### Measurement.

Body length males 3.0–3.2 mm, females 3.2–3.3 mm.

#### Specimen examined.

***Holotype*** ♂: China, Guizhou Prov., Dejiang, 22.VII.2017, coll. Chang Han and Bin Yan, on grape (GUGC). ***Paratypes***: 5♂♂, 5♀♀, same data as holotype.

#### Etymology.

The new species is named from the Latin word “*furcatus*”, referring to the forked aedeagal process.

#### Remarks.

The new species is similar to Arboridia (A.) anteoculara Song & Li, 2013, but differs in only having a pair of processes on the ventrobasal surface of aedeagal shaft (Figs 17, 24); the latter species having two pairs of processes and arising from both sides of the aedeagal shaft.

#### Host.

*Vitisvinifera* L. (grape).

### ﻿ Arboridia (Arboridia) rubrovittata

Taxon classificationAnimaliaHemipteraCicadellidae

Han sp. nov.

7F4B4A1C-1668-51A4-A9B0-0B691DD97432

https://zoobank.org/AE69B8DF-EB76-4CB8-A0EA-B9EB4D1FCB43

Figs 5–9 , 29–36 , 37–44

#### Description.

Dorsum orange, yellow or beige. Eyes black, inner and posterior margins white (Figs 5, 6). Vertex without pair of dark spots, with a white patch each side of midline basally; coronal suture orange yellow (Figs 5, 7, 8). Face orange yellow. Pronotum with ivory or pale white streaks situated laterad of anterior margin. Scutellum pale or whitish yellow with lateral triangles dark to pale brown (Figs 5, 7, 8). Forewing with oblique pale reddish-orange vittae in clavus and adjacent area of wing. Abdominal segments milky yellow, tergites with segment margins brown. Subgenital plate with nearly 2/3 apically dark (Figs 5, 6).

Ventral abdominal apodemes small, extended to posterior margin of 3^rd^ sternite (Figs 32, 43).

**Figures 29–36.**
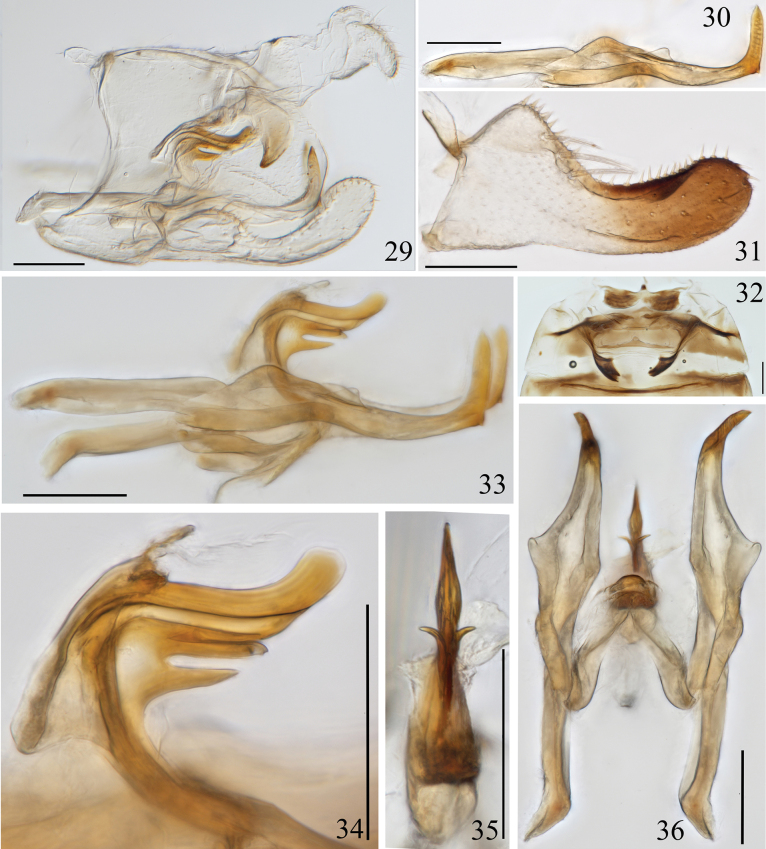
Arboridia (Arboridia) rubrovittata Han, sp. nov. **29** male genitalia, lateral view **30** style, lateral view **31** subgenital plate **32** abdominal apodemes **33** aedeagus, connective and style, lateral view **34** aedeagus, lateral view **35** aedeagus, ventral view **36** aedeagus, connective and style, ventral view. Scale bars: 0.1 mm.

**Figures 37–44.**
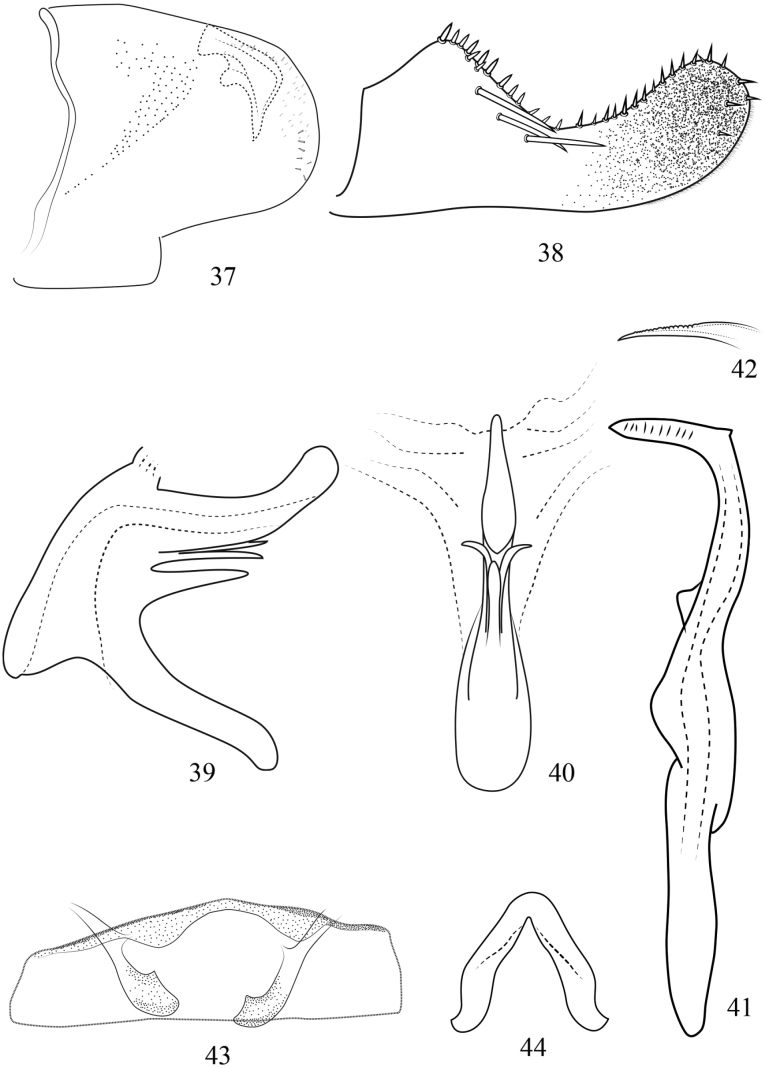
Arboridia (Arboridia) rubrovittata Han, sp. nov. **37** male pygofer, lateral view **38** subgenital plate **39** aedeagus, lateral view **40** aedeagus, ventral view **41** style, lateral view **42** style apex, ventral view **43** abdominal apodemes **44** connective.

***Male genitalia*.** Pygofer dorsal appendage claw-like (Figs 29, 37). Subgenital plate with 3 lateral macrosetae in an oblique row slightly basad of midlength (Figs 31, 38). Style slender, with 2 points, heel point small; sword-like apically with transverse wrinkles in lateral view (Figs 30, 41), serrated in ventral view (Fig. 42). Aedeagus relatively small, shaft laterally compressed, digitate and slightly upturned in lateral view; subbasally with three processes, two basal processes and a single unpaired spike basad, the distal paired processes divergent with branches slender, the proximal process slightly shorter and more robust, finger-like in ventral view (Figs 34, 35, 39, 40); preatrium long. Connective V-shaped (Figs 36, 44).

#### Measurement.

Body length males 2.7–2.9 mm, females 2.9–3.0 mm.

#### Specimen examined.

***Holotype*** ♂: China, Guizhou Prov., Jianhe, 26.V.2017, coll. Chang Han and Yaowen Zhang, on kiwi (GUGC). ***Paratypes***: 23♂♂, 25♀♀, same data as holotype on kiwi; 3♂♂, 6♀♀, China, Guizhou Prov., Wujiang, 19.V.2017, coll. Chang Han and Bin Yan, on walnut (GUGC).

#### Etymology.

The new species name is derived from the Latin words “*ruber*” (red) and “*vittatus*” (banded), referring to the reddish-orange oblique stripes on the forewings.

#### Remarks.

The new species can be distinguished from most *Arboridia* species by its vertex and pronotum without dark spots (Figs 5, 7, 8) and reddish-orange stripes on the forewing. Its male genitalia is similar to A. (A.) lunula Song & Li, 2013, but can be distinguished by the sword-like apex of the style and aedeagus with three basal ventral processes, the upper paired processes slender (Figs 34, 39).

#### Host.

*Actinidiachinensis* Planch (kiwi fruit); *Juglansregia* L. (walnut).

### ﻿ Arboridia (Arboridia) robustipenis

Taxon classificationAnimaliaHemipteraCicadellidae

Han sp. nov.

5A42B725-4A69-5511-9D13-A52A903975AB

https://zoobank.org/C2F0CB93-2BA0-498A-878F-F91A7FE2D8DD

Figs 10–13 , 45–52 , 53–59

#### Description.

Head with eyes black with posterior margin pearl white; crown yellow with a dark yellow spot at apex, an adjacent brown spot posteriorly on each side of midline and a brown patch at base of coronal suture (Figs 10–12). Face pale yellow, with anteclypeus brown apically; gena whitish yellow (Fig. 13). Pronotum semitransparent with brown markings (Fig. 12). Scutellum yellow with lateral triangles dark brown (Fig. 12). Forewing brownish hyaline with off-white patch in clavus and brochosome region. Abdominal segments dark brown, sternites with yellow hind margins of segments; subgenital plates black apically (Figs 10, 11).

Abdominal apodemes small, not exceeding posterior margin of 3^rd^ sternite (Figs 49, 58).

**Figures 45–52.**
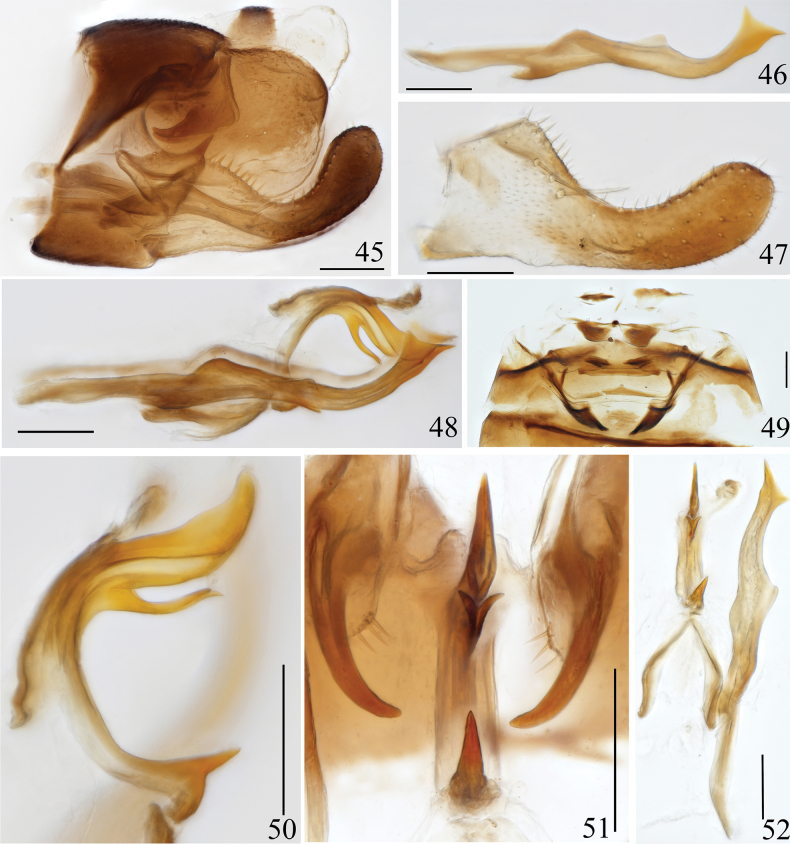
Arboridia (Arboridia) robustipenis Han, sp. nov. **45** male genitalia, lateral view **46** style, lateral view **47** subgenital plate **48** aedeagus, connective and style, lateral view **49** abdominal apodemes **50** aedeagus, lateral view **51** aedeagus & pygofer dorsal appendage, ventral view **52** aedeagus, connective and style, ventral view. Scale bars: 0.1 mm.

**Figures 53–59.**
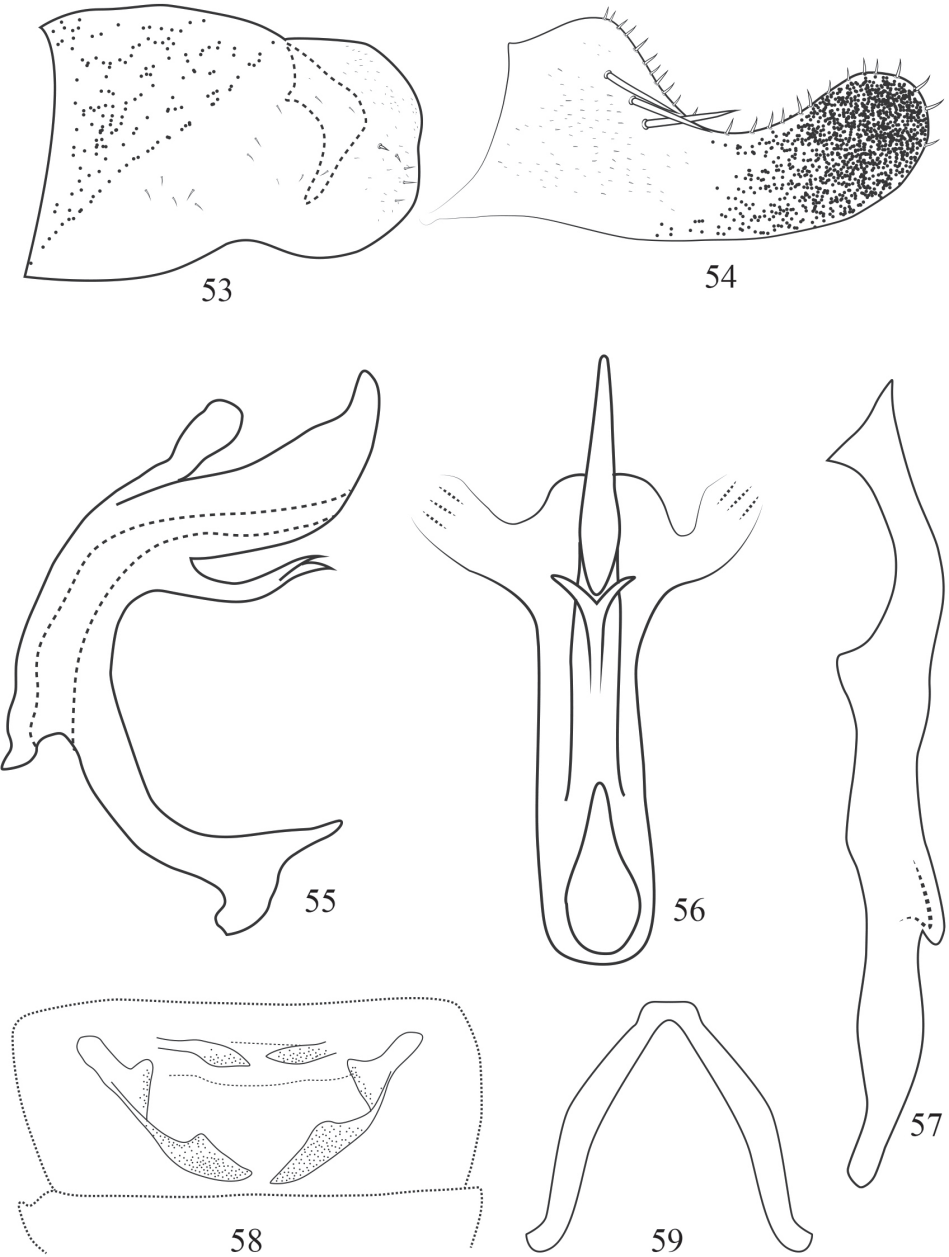
Arboridia (Arboridia) robustipenis Han, sp. nov. **53** male pygofer, lateral view **54** subgenital plate **55** aedeagus, lateral view **56** aedeagus, ventral view **57** style, lateral view **58** abdominal apodemes **59** connective.

***Male genitalia*.** Pygofer dorsal appendage tapering and curved ventrad (Figs 45, 53). Subgenital plate with 3 lateral macrosetae in an oblique row slightly basad of midlength (Figs 47, 54). Style apex with two triangular points; preapical lobe well developed (Figs 46, 57). Aedeagal shaft strongly laterally compressed and “C” shaped with apex upturned in lateral view, with two basal processes fused for 2/3 of their length at midlength of ventral margin; preatrium long, with a thorn-like basal projection (Figs 50–51, 55–56). Connective V-shaped with stem reduced (Figs 52, 59).

#### Measurement.

Body length males 2.9–3.2 mm, females 3.0–3.3 mm.

#### Specimen examined.

***Holotype***: ♂, China, Guizhou Prov., Wujiang, 19.V.2017, coll. Chang Han and Bin Yan, on walnut (GUGC). ***Paratypes***: 5♂♂, 7♀♀, same data as holotype on walnut; 50♂♂61♀♀, China, Guizhou Prov., Xiuwen, 19.VII.2017, coll. Chang Han and Bin Yan, on kiwi (GUGC)

#### Etymology.

The new species name is derived from the Latin words “robustus” and “penis”, and refers to the robust aedeagal shaft in lateral view.

#### Remarks.

The new species can be distinguished from A. (A.) luojiashangensis Zhang & Song, 2022 by the aedeagus with strongly laterally compressed shaft “C” shaped; the paired basal processes fused for 2/3 of their length like a forked tongue (Figs 50–51, 55–56); and the preatrium with a thorn-like basal projection.

#### Host.

*Actinidiachinensis* Planch (kiwi fruit); *Juglansregia* L. (walnut).

## Supplementary Material

XML Treatment for Arboridia

XML Treatment for Arboridia (Arboridia) furcata

XML Treatment for Arboridia (Arboridia) rubrovittata

XML Treatment for Arboridia (Arboridia) robustipenis

## ﻿Additional information

### Author ORCIDs

Bin Yan https://orcid.org/0000-0002-5290-4576

Maofa Yang https://orcid.org/0000-0002-5523-6825

Michael D. Webb https://orcid.org/0000-0000-0000-0000

### Data availability

All of the data that support the findings of this study are available in the main text.

## References

[B1] Cao YH Dmitriev DA Dietrich CH Zhang YL (2019) New taxa and new records of Erythroneurini from China (Hemiptera: Cicadellidae: Typhlocybinae). Acta Entomologica Musei Nationalis Pragae 59(1): 189–210. 10.2478/aemnp-2019-0017

[B2] Dietrich CH (2005) Keys to the families of Cicadomorpha and subfamilies and tribes of Cicadellidae (Hemiptera: Auchenorrhyncha). The Florida Entomologist 88(4): 502–517. 10.1653/0015-4040(2005)88[502:KTTFOC]2.0.CO;2

[B3] Dmitriev DA Anufriev GA Bartlett CR Blanco-Rodríguez E Borodin OI Cao YH Deitz LL Dietrich CH Dmitrieva MO El-Sonbati SA Evangelista de Souza O Gjonov IV Gonçalves AC Hendrix S McKamey S Kohler M Kunz G Malenovský I Morris BO Novoselova M Pinedo-Escatel JA Rakitov RA Rothschild MJ Sanborn AF Takiya DM Wallace MS Zahniser JN (2022) [onward] World Auchenorrhyncha Database. TaxonPages. https://hoppers.speciesfile.org [Retrieved on 2024-02-21]

[B4] Dworakowska I (1972) On some Oriental Erythroneurini (Auchenorrhyncha, Cicadellidae, Typhlocybinae). Bulletin de l’Académie Polonaise des Sciences. Série des Sciences Biologiques 20(6): 395–405.

[B5] Dworakowska I (1993) Remarks on *Alebra* Fieb. And Eastern Hemisphere Alebrini (Auchenorrhyncha: Cicadellidae: Typhlocybinae). Entomotaxonomia 15(2): 91–121.

[B6] Dworakowska I Viraktamath CA (1975) On some Typhlocybinae from India (Auchenorrhyncha, Cicadellidae). Bulletin de l’Académie Polonaise des Sciences. Série des Sciences Biologiques 23(8): 521–530.

[B7] Jiang J Luo GM Song YH (2021) Two new species of the genus *Arboridia* Zachvatkin from China (Hemiptera: Cicadellidae: Typhlocybinae). Zootaxa 3(3): 349–357. 10.11646/zootaxa.5005.3.9 34811254

[B8] Matsumura S (1932) A revision of the Palaearctic and Oriental Typhlocybid-genera with descriptions of new species and new genera. Insecta Matsumurana 6(3): 93–120.

[B9] Pu TY Wang JQ Song YH (2023) Two new species of Erythroneurini from China (Hemiptera: Cicadellidae: Typhlocybinae). Zootaxa 5374(2): 295–300. 10.11646/zootaxa.5374.2.8 38220858

[B10] Sohi AS Sandhu PK (1971) Arborifera - a new subgenus of Arboridia Zachv. (Typhlocybinae, Cicadellidae) from Punjab, India, with description of its immature stages. Bulletin de l’Académie Polonaise des Sciences. Série des Sciences Biologiques 19(6): 401–406.

[B11] Song YH Li ZZ (2013) Some new species and new record of the genus *Arboridia* Zachvatkin (Hemiptera: Cicadellidae: Typhlocybinae) from six provinces of China. Zootaxa 3613(3): 229–244. 10.11646/zootaxa.3613.3.2 24698914

[B12] Song YH Li ZZ Dietrich CH (2016) Contribution to knowledge of Typhlocybinae (Hemiptera: Cicadellidae) in Thailand: first record of genus *Arboridia* Zachvatkin with description of three new species and a new synonymy. Zootaxa 4171(2): 357–364. 10.11646/zootaxa.4171.2.8 27701229

[B13] Zachvatkin AA (1946) Studies on the Homoptera of Turkey. I–VII. Transactions of the Royal Entomological Society of London 97(6): 149–176. 10.1111/j.1365-2311.1946.tb00278.x

[B14] Zhang N Jiang J Song YH (2022) Two new species of the genus *Arboridia* Zachvatkin from karst area of southwestern China (Hemiptera: Cicadellidae: Typhlocybinae). Journal of Asia-Pacific Entomology 25(3): 101970. 10.1016/j.aspen.2022.101970

